# Multiple-Phase Biometric Relationships and Sexual Maturity in the Atlantic Bluefin Tuna, *Thunnus thynnus* (Osteichthyes: Scombridae)

**DOI:** 10.3390/ani11020390

**Published:** 2021-02-03

**Authors:** Giambattista Bello, Nicoletta Santamaria, Aldo Corriero

**Affiliations:** 1 Independent Researcher, 70042 Mola di Bari, Italy; 2 Department of Emergency and Organ Transplantation, Section of Veterinary Clinics and Animal Production, University of Bari Aldo Moro, 70010 Valenzano, Italy; nicoletta.santamaria@uniba.it (N.S.); aldo.corriero@uniba.it (A.C.)

**Keywords:** growth stanzas, change points, biometric correlations, sexual maturity

## Abstract

**Simple Summary:**

Fish undergo distinct growth phases during their life. Passages from one growth phase to the following one correspond to a dramatic change in body proportion and/or growth rate. These changes can be detected as change-points in biometric relationships, such as that between length and weight. In this paper, we checked whether any change-points could be detected in the somatic proportions of the Atlantic bluefin tuna during its growth in order to better define some life history traits, including size at sexual maturity, that represent the essential basic knowledge for the effective management of this species. Fork length–weight relationship, length–age relationship (von Bertalanffy growth equation), and the relationship between the surface of the cross section of the first dorsal spine (a measure indicative of spine bone thickness) and the fork length were examined. All of the somatic relationships showed a change-point between 101 and 110 cm fork length. The present results corroborate the disputed hypothesis that Atlantic bluefin tuna from the eastern stock reproduce for the first time at 3–4 years of age.

**Abstract:**

Most fish undergo distinct growth phases during ontogenesis. An extremely important passage from the juvenile to adult phase occurs at the onset of sexual maturity, which shows in body proportion and/or growth rate changes. These can be detected as change-points in biometric relationships. In this paper, the Atlantic bluefin tuna was analyzed to verify whether its somatic proportions show any sign of discontinuity during growth, i.e., whether any change-points may be detected in its somatic proportions. This fish has never been examined in this respect, and single-phase models, which are indeed easier to both compute and apply, are used in stock analyses. The following somatic relationships were analyzed in Atlantic bluefin tuna captured in the Mediterranean Sea between 1998 and 2010: “fork length–weight” regression, the von Bertalanffy growth equation, and “first dorsal spine cross section surface–fork length” regression. All of the examined relationships were found to be best modelled by multiple-phase regression equations, and all of them showed a change-point within the range of 101–110 cm fork length, which corresponds to 3–4 years of age. The present results, based on reproductive state-independent analyses, corroborate the disputed hypothesis that Atlantic bluefin tuna from the eastern stock in fact reproduce for the first time at this age.

## 1. Introduction

As early as the middle of the last century, Vasnetsov [1], referring to early ontogenetic phases, introduced the concept of stanzas of growth, i.e., distinct phases in the life cycle of fish between which rather abrupt changes in physiology induce changes in growth patterns. The passage from one growth stanza to the following one may become evident as a change in body form, which shows up in the length–weight relationship, or as a sudden change in the growth rate [2]. Indeed, such a change may affect morphometric correlations among different anatomical structures, as well as to the correlation between body weight and length [3,4]. In addition to the passage from one growth stanza to the following one in fish early ontogenesis [1], another important passage occurs at the onset of sexual maturity [4,5,6,7].

In general, it may be assumed that the ontogenetic passage through growth stanzas involves the vast majority of, if not all, fish. In this respect, Froese [6] stressed that the adequate description of all growth phases of a fish is essential in order to assess animal overall growth patterns and to model aquatic ecosystems for fishery purposes. Moreover, knowledge of the precise growth parameters is necessary in order to assess the spawning biomass, productivity, and sustainable yield of exploited stocks [8]. Conversely, according to the literature [9], the use of an incorrect growth model can affect the estimated age structure of the population, and might bias the fishing mortality and biomass estimate (as stated by the authors of [10], some fish growth may be modelled by two- or three-stanza curves).

The detection of modifications in length–weight regressions and/or growth equations during fish growth, i.e., passages from one growth stanza to the next, may contribute to discerning the onset of sexual maturity when other methods are not implementable [11]. Moreover, the determination of sexual maturity by gonad observations and the recognition of modifications in biometric relationships may validate each other by attesting the occurrence of maturity in fish.

Despite the above facts and recommendations, presently, the computation of single-phase equations for both length–weight regression and growth curves for fish is still a common practice [12,13]. The use of single-phase models is possibly fostered by their ease of computation, as well as application to subsequent level models (e.g., stock analyses). This also applies to the Atlantic bluefin tuna (ABFT), *Thunnus thynnus* (Linnaeus, 1758) (Osteichthyes: Scombridae), an important and widely studied halieutic species, whose length–weight relationship and growth curve have always been modelled by single equations throughout its size range (e.g., for length–weight relationships [14,15,16] and for growth equations [14,17,18]).

The adoption of appropriate biometric models holds notable importance in the case of ABFT, as its age-at-reproduction is presently a matter of debate [19,20]. The International Commission for the Conservation of Atlantic Tunas (ICCAT) manages ABFT on the assumption that the two recognized stocks, i.e., the Western and the Eastern Atlantic stocks, have different sexual maturation schedules and strict spawning fidelity to the respective reproductive areas (Gulf of Mexico and the Mediterranean Sea) [21]. This disputed assumption is backed by controversial determinations of main life traits, such as age- and size-at-sexual maturity [19,20]. In this respect, any piece of evidence able to shed light on the age- and size-at-first reproduction is highly welcome.

The purpose of this paper is to verify the occurrence of change-points (or inflection-points or break-points; i.e., points of discontinuity in regression slopes), if any, in three biometric relationships, namely “fork length–body weight”, “fork length–age”, and “surface of first dorsal spine cross section–fork length”, for juvenile and adult ABFT caught in the Mediterranean, as the expression of the existence of multiple growth stanzas. The specific aim of this study is to detect any correspondence between change-points and sexual maturity in order to better define the life history traits that represent the base of knowledge necessary for effective management of this species.

## 2. Materials and Methods

### 2.1. Data Sources

The data sets on which the present paper is based have been used in previous studies of ours [14,22]. The examined ABFT specimens were collected from 1998 to 2010 (all months were sampled, except January and February), in the following areas of the Mediterranean Sea: South Adriatic Sea, South Tyrrhenian Sea, North Ionian Sea, and Ionian waters around Malta. In all, data from 375 (198 males and 177 females) ABFT specimens were employed for the present computations. In addition, data from three males and one female were found to be outliers [23] and were discarded. In detail, data from all specimens were used to analyze the fork length–weight relationship for each sex and a subset of 186 specimens for the age–fork length correlation (growth equation) and the spine cross section surface–fork length regression. As for the growth equation, we used the age estimates from [22] that particularized age into two sub-year units, i.e., 1.25, 1.75, 2.25, 2.75, and so on, up to the seventh year, which showed a finer descriptive precision. To model the growth of the first spine of the first dorsal fin with respect to the animal growth in length, we used the surface of a cross section of the spine (details on how the sectioning plan was determined are provided in [22]), as it was found to be a better descriptor than the spine diameter. In both latter correlations, data from males and females were pooled, as no significant differences were found between the two sexes [22].

### 2.2. Measurement Abbreviations and Symbols

The following abbreviations were adopted: *FL* = fork length; *W* = body mass; *SS* = surface of the cross section of the first dorsal spine at the distance of half the maximum spine diameter from the condyle base [22]; *FLWR* = fork length–body mass correlation; *VB* = von Bertalnffy growth equation (fork length–age relationship); *SSFLR* = first dorsal spine cross section surface–fork length relationship.

The following two symbols need to be explained, because they might be mistaken for each other: *k* is the *n_i_* specimen of first phase in the likelihood ratio test by Quandt, and *K* is the growth coefficient in the von Bertalanffy growth curves (see further).

### 2.3. Data Analyses

To better describe discontinuities in the relationships between the pairs of biometric parameters, hence the different growth stanzas, the two-segment model was applied, because it was reported to be the best one, among several, to describe such discontinuities in fish [4].

For the purpose of detecting change-, inflection-, or break-points, i.e., the above-mentioned discontinuities, the regression curves for *FLWR*, *VB*, and *SSFLR* were transformed into linear ones in order to adequately analyze them. To linearize the *FLWR* and *SSFLR* curves, which are best described by the allometric or power equation model *W* = *aL^b^*, the data were log-transformed (natural logarithms) and fitted to a linear equation. As for the *VB*, the data were fitted to the Ford–Walford model, whose plot renders a linear correlation [2]. Then, the residuals of all equations were computed and their distributions were analyzed in order to both spot the occurrence of outliers and remove them [23], and to reveal possible departures from the linearity of their respective regression lines. Hence, the hypothesis of a two-phase regression was tested using the likelihood ratio test by Quandt (Q-test) [24] according to the two-segment model, which is canonically used to describe two-phase length–weight correlations [4]. When evidence for a three-phase regression came out, the likelihood ratio test, after detecting the first change-point, was applied once again to the second phase regression in order to detect the second change-point.

Following the detection of the change-point(s) in each correlation, the regression equations for the pairs of phases (in two-phase regressions, “small” vs. “large” specimens, and in three-phase regression, “small” vs. “medium” and “medium” vs. “large” specimens) were examined and their slopes were compared using Student’s t-test to verify any significant differences.

## 3. Results

### 3.1. Fork Length–Body Mass Relationship (FLWR)

**Figure 1:**
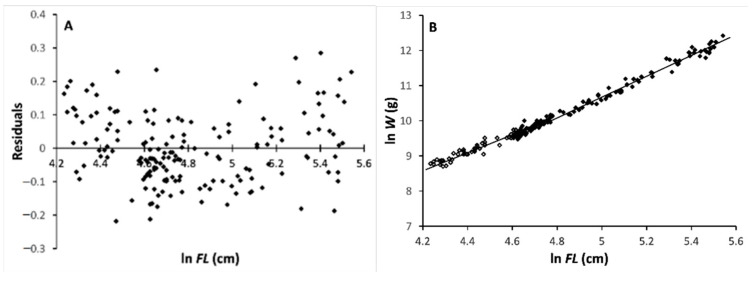
Fork length–body mass relationship (*FLWR*) for male Atlantic bluefin tuna (ABFT). (**A**) Residual body mass (ln-transformed *W*) distribution vs. fork length (ln *FL*). (**B**) Double-logarithmic plot of body mass (W) vs. fork length (FL) for the two development phase, juveniles and adults.

**Figure 2:**
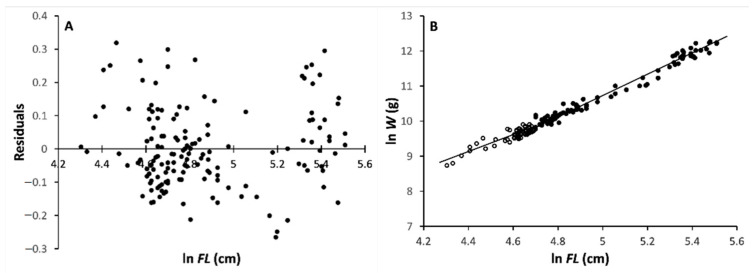
Fork length–body mass relationship (*FLWR*) for female ABFT. (**A**) Residual body mass (ln-transformed *W*) distribution vs. fork length (ln *FL*). (**B**) Double-logarithmic plot of body mass (*W*) vs. fork length (*FL*) for the two development phase, juveniles and adults.

The residuals of both males and females were distributed according to a V-shape (Figure 1A and Figure 2A), with the second branch slope being higher than the first one. This suggested the possible occurrence of an inflection in the *FLWR* line.

The likelihood ratio test applied to the (ln *FL*, ln *W*)_i_ data also showed a departure from the linearity of their distributions, i.e., the occurrence of a two-phase regression, in both sexes.

In males, the minimum value of the likelihood ratio was found at *k* = 65, which corresponds to a specimen with *FL* = 101 cm. The regression equations (Figure 1B) for the first *k* males (“small”) and for the remaining (*n – k*) males (“large”) were, respectively:

ln *W_s_* = –1.379 + 2.388 ln *FL_s_* (*n* = 65; size range: 69–101 cm *FL*; *s_b_* = 0.091; *r* = 0.957) (1)

ln *W_l_* = –3.722 + 2.884 ln *FL_l_* (*n* = 133; size range: 102–255 cm *FL*; *s_b_* = 0.029; *r* = 0.994). (2)

The slopes of the two equations, *b_s_* and *b_l_*, were found to be very highly significantly different (*t_slope_* = 5.178; *df* = 194; *P_t_* = 2.8^–7^). In both equations, the slope values for *b* were considerably < 3.

As for females, the minimum value of the likelihood ratio was found at *k* = 73, which corresponds to a specimen with *FL* = 110 cm. Incidentally, when the minimum value of the likelihood ratio was found to generate an unrealistic result, i.e., *b_l_* >> 3, it was discarded; in general, the *FLWR* slope values for *b* were well below 3. The regression equations (Figure 2B) for the first *k* females (“small”) and for the remaining (*n – k*) females (“large”) were, respectively:

ln *W_s_* = –0.919 + 2.282 ln *FL_s_* (*n* = 73; size range: 74–110 cm *FL*; *s_b_* = 0.130; *r* = 0.901) (3)

ln *W_l_* = –3.862 + 2.918 ln *FL_l_* (*n* = 104; size range: 110–247 cm *FL*; *s_b_* = 0.045; *r* = 0.988). (4)

The slopes of the two equations, *b_s_* and *b_l_*, were found to be significantly different (*t_slope_* = 4.620; *df* = 173; *P_t_* = 3.7^–6^). In both equations, the slope values for *b* were <3.

Therefore, the change-point separating the *FLWR* equations into two distinct phases was between 101 and 102 cm FL, and at 110 cm *FL* for males and females, respectively. In both sexes, after the change in slope, the weight growth rate increased with respect to the length growth rate, that is d(*W_l_*/*L_l_*)/d*t* > d(*W_s_*/*L_s_*)/d*t*, where *t* is time and d is differential. In all of the cases, the slope value of *b* was significantly < 3, indicating that the growth in weight with respect to that in length is negatively allometric in both phases for both sexes.

### 3.2. Growth in Length (VB)

**Figure 3:**
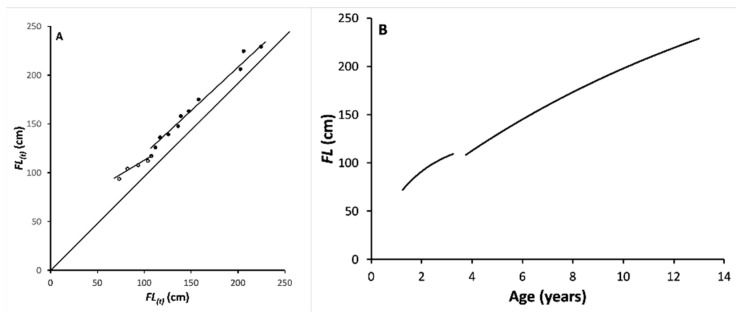
Growth in fork length for pooled male and female ABFT. (**A**) Ford–Walford plot. (**B**) Von Bertalanffy growth curves for the two development phases—juveniles and adults.

According to the likelihood ratio test applied to the Ford–Walford plot (Figure 3A), there was an inflection in the von Bertalanffy growth curve (data from males and females pooled), thus showing the existence of two-phases in the ABFT growth in length.

The minimum value of the likelihood ratio was found at *k* = 5, which corresponds to the 3.25-year-old group. The regression equations for the first *k* age groups (small) and for the remaining (*n – k*) age groups (large) were, respectively:

small: *FL*_(*t*+1)_ = 51.739 + 0.598 *FL*_(*t*)_ (*n* = 5; age range: 1.25–3.25 y (corresponding mean FL range: 73.4–107.7); *s_b_* = 0.084; *r* = 0.971) (5)

large: *FL*_(*t*+1)_ = 29.691 + 0.923 *FL*_(*t*)_ (*n* = 10; age range: 3.75–13 y (corresponding mean FL range: 112.1–229.1); *s_b_* = 0.042; *r* = 0.992). (6)

The slopes of the two equations, *b_s_* and *b_l_*, were found to be highly significantly different (*t_slope_* = 3.449; *df* = 11; *P_t_* = 2.7^–3^). Therefore, the ABFT growth in length is best modelled by two equations, corresponding to two developmental phases, before and after the change-point that occurs between the ages of 3.25 and 3.75 years.

The parameters of the corresponding von Bertalanffy growth equations (Figure 3B) are the following:

small: *L_inf_* = 126.56 cm *FL* (*se* = 11.60); *K_s_* = 0.57 (*se* = 0.20); *t_0_* = −0.22 (*se* = 0.25) (7)

large: *L_inf_* = 360.94 cm *FL* (*se* = 14.75); *K_l_* = 0.07 (*se* = 0.01); *t_0_* = −1.33 (*se* = 0.18). (8)

They clearly show that growth is significantly slowed down in the second developmental phase (*K_l_* << *K_s_*).

### 3.3. First Dorsal Spine Cross Section Surface—Fork Length Relationship (SSFLR)

**Figure 4:**
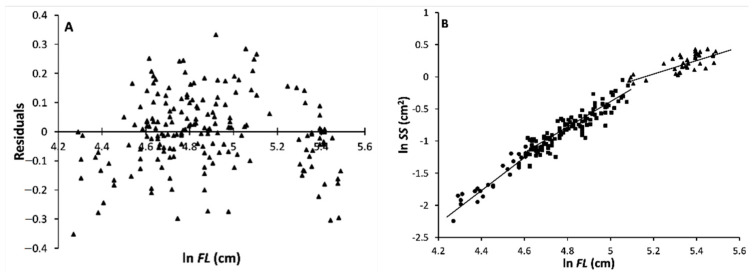
First dorsal spine cross section surface–fork length relationship (*SSFLR*) for pooled male and female ABFT. (**A**) residual first dorsal spine cross section surface (ln-transformed *SS*) distribution vs. fork length (ln *FL*); (**B**) double-logarithmic plot of the first dorsal spine cross section surface (*SS*) vs. fork length (*FL*) for the three detected phase, juveniles (up to 7 years of age) and adults (older than 7 years old).

As for the regression of the surface of the first dorsal spine cross section, *SS*, on *FL*, both the residual distribution (И-shaped) and the likelihood ratio test showed a double departure from linearity in the distribution of the (ln *SS*, ln *FL*)*_i_* data (data from males and females are pooled). Hence, this is a three-phase regression. The inflection points were found at *k_1_* = 29 (FL = 101 cm) and *k_2_* = 144 (FL = 161 cm; Figure 4).

The equations for the three growth stanzas were as follows:

ln *FL_s_* = –13.087 + 2.587 ln *SS_s_* (*n* = 29; size range: 71.5–101 cm *FL*; *s_b_* = 0.163; *r* = 0.950) (9)

ln *FL_m_* = –10.509 + 2.019 ln *SS_m_* (*n* = 115; size range: 101–161 cm *FL*; *s_b_* = 0.086; *r* = 0.910) (10)

ln *FL_l_* = –5.567 + 1.082 ln *SS_l_* (*n* = 40; size range: 161–242 cm *FL*; *s_b_* = 0.146; *r* = 0.769) (11)

The slopes of both the first and second pairs of equations (*b_s_* – *b_m_* and *b_m_* – *b_l_*, respectively) were found to be highly significantly different:

*b_s_* vs. *b_m_*: *t_slope_* = 3.027; *df* = 140; *P_t_* = 1.5^–3^ (12)

*b_m_* vs. *b_l_*: *t_slope_* = 5.525; *df* = 151; *P_t_* = 7.1^–8^ (13)

In the first equation (small specimens), the slope value, *b_s_*, was significantly > 2; in the third one (large specimens), *b_l_* was significantly < 2; whereas, in the second equation (intermediate specimens), the slope, *b_m_*, was found not to differ significantly from 2.

The length at which the change-points occurred in all of the correlations are summarized in Table 1.

## 4. Discussion

The present results clearly show that all of the examined somatic relationships, i.e., fork length–body mass (*FLWR*) for males and females, fork length–age (*VB*), and first dorsal spine cross section surface–fork length (*SSFLR*), are best modelled by multiple-phase regression equations, with the first two being biphasic and the latter being triphasic. Accordingly, the ABFT goes across at least two different growth stanzas during its life cycle.

Biphasic growth curves have also been detected in other scombrid fish, including *Thunnus* spp. [10], but never in ABFT, despite the intense research that has so far been undertaken worldwide to improve the knowledge of the life cycle of this important fishery resource, which is undergoing an intense fishing effort, is classified as endangered species by the International Union for Conservation of Nature (https://www.iucnredlist.org/species/21860/9331546), and whose fishery is subjected to strict regulations [25,26].

In the present study, a close correspondence among the sizes at change-point for all of the examined regressions was found. All of them fall within the narrow range of 101–110 cm *FL*, corresponding to an estimated age of between 3 and 4 years, in accordance with the length–age key given by the authors of [14]. As for the first dorsal spine cross section surface–fork length relationship, a further change-point at 161 cm *FL*, corresponding to 7 years of age according to the above-mentioned age–length key, was observed.

In all three somatic relationships, the observed change-points (the first one in the *SSFLR*) occur at about the onset of sexual maturation [27,28,29,30]. In other words, the inflection in all regression lines marks the passage from the pre-maturation growth stanza to the adult one. As a matter of fact, there is a growing corpus of evidence that the onset of sexual maturity is detectable in many fish species by changes in the somatic proportions and/or in the growth progress modality. Seemingly, this is a constant feature of fish species. Several authors have used inflections in somatic growth to indirectly determine the achievement of sexual maturity by means of inflections in length-at-age equations [11,31].

Incidentally, it must be pointed out that the computation of single length-weight regressions and/or growth equations instead of two-phase equations is strongly biased by the size composition of the sample [7,10], which contributes to explaining why there are so many considerably different equation parameters in the literature [10,15].

The *FLWR* results show that the slope coefficient is always < 3 in both phases of both sexes, which indicates a negative allometric growth, quite marked in the juvenile stanza (*b* = 2.388 and 2.282 in males and females, respectively) and slightly marked in the adult one (*b* = 2.884 and 2.918 in males and females, respectively). The finding of negative allometric growth is in agreement with most *FLWR* for ABFT available in the literature. As for length growth, the parameter *K* of *VB* noticeably decreased from 0.57 in the first phase to 0.07 in the second phase, showing that growth rates slow down after maturity is reached. Incidentally, the second phase *L_inf_* value is somewhat different from the *L_max_* values reported in the literature [15,17], possibly because of the limited age range data available for the present study, up to 14 years. Putting together these results, it appears that ABFT by far favors growth in length before puberty (*FLWR*: *b* << 3; *VB*: *K_s_* >> *K_l_*), mainly investing its energy in the muscoloskeletal system; whereas, after puberty, energy is largely diverted to the visceral growth, which is related to the storage of energy reserves for gonad development and gamete production. The privileged muscoloskeletal growth in prepubertal life is seemingly aimed at reaching a large size in a comparatively short time, which may be related to escaping predators, thanks to the gain of a long size, which in turn increases swimming speed. In fact, fish speed increases with fish length [32], which therefore contributes to explaining why young ABFT conduct shorter spawning migrations than large adults [33]. Swimming efficiency is particularly relevant for highly migratory species like ABFT, capable of performing trans-oceanic migrations [34,35,36], and body size might affect its capacity to rapidly move between feeding and reproduction grounds [19].

As mentioned above, it is more and more evident that inflections in length–age growth curves and in *LWR* lines are caused by the achievement of sexual maturity in many of the examined fish. In the present case, the significant slope changes discovered in all three of the somatic correlations are most likely related to the same physiological causative agent, that is the onset of sexual maturity, which has been documented to occur at the same size/age of inflection points [27,28,29,30].

The present results contribute to corroborating previous findings about age-at-maturity, an important trait of the ABFT life cycle. Efforts to unmask ABFT size and age at sexual maturity date back to almost one century ago, and have been established on different investigation methods. Based on the controversial outputs of such efforts, the ICCAT fixed the ages for sexual maturity at 8–12 and 3–5 years for Western and Eastern stock, respectively [19]. Although several kinds of methodological approaches—i.e., analysis of seasonal changes of gonad macroscopic appearance and gonad mass/body mass ratio [27,28,29], gonad histological analysis [29,37,38], and biochemical and molecular endocrinological analyses [39,40,41]—converge on the substantiation that ABFT from the Eastern stock reproduce at between 3 and 5 years of age (median length at maturity about 104 cm *FL*) [30], the present age at maturity fixed by ICCAT is still a debated matter. Criticisms regarding the age at maturity of the Eastern ABFT stock proposed by the authors of [30] are based on the fact that biological samplings in the Mediterranean Sea were not representative of the whole stock, because all of the examined fish were caught on spawning grounds during spawning season [19]. The present study is based on biometric data from fish sampled in the Mediterranean Sea throughout the year, and its results are not affected by the reproductive conditions of the sampled fish and, therefore, it represents a further, robust, confirmation of the reported size and age of sexual maturity of ABFT from Eastern stock.

As for the correlation between the first dorsal spine section surface and fork length, *SSFLR*, the results showed that it displays two change-points, hence the development of the spine goes through three phases. Galilei [42] showed that an animal bone cross section surface is proportional to the square of its length. According to this proportion, which we may aptly call the “Galilean allometric relationship”, it is expected that *SS* ∝ *FL*^2^, because the bone cross section surface ∝ bone length^2^ ∝ animal length^2^. Therefore, the growth in width through age of the first dorsal spine is deemed isometric when the regression coefficient of *SS* on *FL* (or slope, *b*, of the *SSFLR*) is 2. Accordingly, *b* = 2.587 in the first phase (71.5–101 cm *FL*; 1–3 years) indicates that the spine growth in width is enhanced with respect to the ABFT body growth in length, i.e., it is positively allometric; in the second phase (101–161 cm *FL*; 3.25–7 years), when *b* = 2.019, the first spine growth in width growth is isometric; whereas in the third phase (161–242 cm *FL*; 7–13 years), *b* = 1.082, the spine width growth is significantly slowed down with respect to body growth. Once again, the first dorsal spine appears to grow fast in juvenile ABFT so as to support the comparatively fast growth in length of the whole body, and after puberty, it becomes regular, i.e., isometric, at least until the age of 7 years/60 cm *FL*. The resulting slowed down growth in the width of the spine seems to imply that, at about 160 cm *FL*, the spine has attained an overall width sufficient to mechanically support the ABFT movements, which need only comparatively small increases in width; in other words, at the age of about 7 years old, the first dorsal spine reaches a new equilibrium with respect to the isometric growth phase.

The first dorsal spine of ABFT (and supposedly also the other fin spines in this, as well as in other tuna species) is a very dynamic organ, whose growth is dependent on seasonal and environmental conditions [14,22]. Fin spines play an important role in the movements of fish [43,44], whose role might be even more important in fast and long-swimming pelagic fishes, such as the ABFT (in addition to acting as mineral reserve organs) [22]. The discovery of a three-phase growth in the width of the ABFT first dorsal spine motivates new questions about its role in this fish’s movement mechanics, which deserve to be answered with ad hoc studies.

## 5. Conclusions

The present study confirms that the ABFT maturity is attained at a size of about 101–110 cm *FL* and 3–4 years of age, that is the size and age corresponding to the inflections in the three examined correlations between the somatic parameters. We have to point out, however, that what we considered to be the first stanza, or juvenile phase, might indeed be a second stanza, as we may reasonably suppose the existence of a larval–early juvenile stanza that precedes the juvenile one. Lastly, the biological explanation of the unexpected second change-point (at 7 years of age) of the first dorsal spine cross section surface on fork length relationship deserves further investigation.

## Figures and Tables

**Table 1:** Atlantic bluefin tuna fork length and age where change-points occur in the analyzed biometric relationships.

Correlation	Sex	Size of *k_i_* Specimen [*FL*, cm]	Estimated Age of *k_i_* Specimen [Years]
*FLWR*	males	101	3 *
*FLWR*	females	110	between 3 and 4 *
*VB*	pooled	108 **	3.25
*SSFLR*	pooled	101 and 161	3 * and 7 *

*k_i_ =* specimen at which change-point occurs; * estimated age at length, after Santamaria et al. (2009); ** mean *FL* corresponding to 3.25 years of age, after Santamaria et al. (2015).

## Data Availability

The authors retain the right to use the data for further elaborations and new publications. Therefore, the data were made available for peer-review only, and might be provided for fishery management or research purposes upon motivated request.
